# Preventing Airborne Disease Transmission: Review of Methods for Ventilation Design in Health Care Facilities

**DOI:** 10.4061/2011/124064

**Published:** 2011-11-15

**Authors:** Amir A. Aliabadi, Steven N. Rogak, Karen H. Bartlett, Sheldon I. Green

**Affiliations:** ^1^Department of Mechanical Engineering, University of British Columbia, 122-6250 Applied Science Lane, Vancouver, BC, Canada V6T 1Z4; ^2^Department of Mechanical Engineering, University of British Columbia, 103-6250 Applied Science Lane, Vancouver, BC, Canada V6T 1Z4; ^3^School of Population and Public Health, University of British Columbia, 364-2206 East Mall, Vancouver, BC, Canada V6T 1Z4; ^4^Department of Mechanical Engineering, University of British Columbia, 2058-6250 Applied Science Lane, Vancouver, BC, Canada V6T 1Z4

## Abstract

Health care facility ventilation design greatly affects disease transmission by aerosols. The desire to control infection in hospitals and at the same time to reduce their carbon footprint motivates the use of unconventional solutions for building design and associated control measures. This paper considers indoor sources and types of infectious aerosols, and pathogen viability and infectivity behaviors in response to environmental conditions. Aerosol dispersion, heat and mass transfer, deposition in the respiratory tract, and infection mechanisms are discussed, with an emphasis on experimental and modeling approaches. Key building design parameters are described that include types of ventilation systems (mixing, displacement, natural and hybrid), air exchange rate, temperature and relative humidity, air flow distribution structure, occupancy, engineered disinfection of air (filtration and UV radiation), and architectural programming (source and activity management) for health care facilities. The paper describes major findings and suggests future research needs in methods for ventilation design of health care facilities to prevent airborne infection risk.

## 1. Introduction

The spread of infectious disease is of global concern for social and economic reasons. For example, seasonal influenza kills 200–500 thousand people annually. In 2009-2010, influenza A (H1N1) caused 17,000 deaths worldwide, many among whom were healthy adults [1, 2]. In 2002-2003, severe acute respiratory syndrome (SARS) killed more than 700 people and spread into 37 countries causing a cost of $18 billion in Asia [2–5]. These recent outbreaks remind us of the potential for a pandemic such as the Spanish flu of 1918–1920 which killed 50–100 million people [5].

Diseases can spread wherever people have direct or indirect contact, but this paper focuses on infections that occur in health care facilities, because they often contain a large proportion of infectious or vulnerable people, and because governments and other health care providers have a clear responsibility to mitigate infections that occur within their walls.

Human-human transmission of disease can result from direct contact with an infected person or an indirect contact through an intermediate object. A direct contact infection could be caused by caregivers not washing hands prior to attending patients [6]. Another common direct contact transmission is due to large infectious aerosols that travel a short distance from the source to the receptor. An important mode of indirect contact is airborne transmission occurring via the spread of fine aerosols, skin flakes, and fungal spores in room air over long distances and time scales. Aerosols can be generated and released by human expiratory actions (speech, coughing, and sneezing), skin shedding, or resuspension from surfaces [7].

Aerosol disease transmission is known to be the main route for many diseases such as *Tuberculosis* and *Aspergillosis*. Also, recent research has shown that the importance of aerosol infection is underrated for common diseases such as influenza, especially during cold and dry seasons [8]. For example, modern experimental techniques have detected infectious aerosols produced by infected patients while breathing, coughing, or sneezing [1]. As far as building ventilation design is concerned, the greatest impact of any ventilation design would be on the airborne route of infection, which is the focus of this paper.

Infection control involves blocking any stage of the infection pathway. For airborne transmission, this can mean reducing the generation of pathogens from an infectious person, using disinfection techniques to kill pathogens released to the air, or simply isolating infectious people in special rooms. Controls generally fall into three categories: administrative, personal protection, and environmental and engineering. Administrative controls aim to keep infectious people away from vulnerable people (infection detection, triage, communication, and education) and ensure that technical controls (e.g., engineering and personal protection) are used correctly. For the airborne transmission pathway, personal protection consists of some form of mask or respirator to prevent either the distribution or inhalation of pathogens [5]. Engineering and environmental controls primarily intervene after pathogens leave the breathing zone from one person before they enter the breathing zone of another.

At the simplest level, an engineering control might involve an increase in room ventilation rates. This would normally decrease pathogen concentrations, which would be expected to reduce infections. However, rooms are not well-mixed, people do not breath in all parts of the room, and pathogen infectivity changes with time and environmental conditions. Furthermore, increased ventilation is not free, because it normally requires larger and more energy intensive equipment. How much should ventilation rates be increased? Which type of system is most helpful in reducing airborne infections? These questions cannot be answered without quantitative estimates of infection risk or at least the relative risk of one engineering system compared to another. Such risk models must include every process during infection from source to receptor. As a result, the entire infection pathway must be considered.

**Figure 1:**
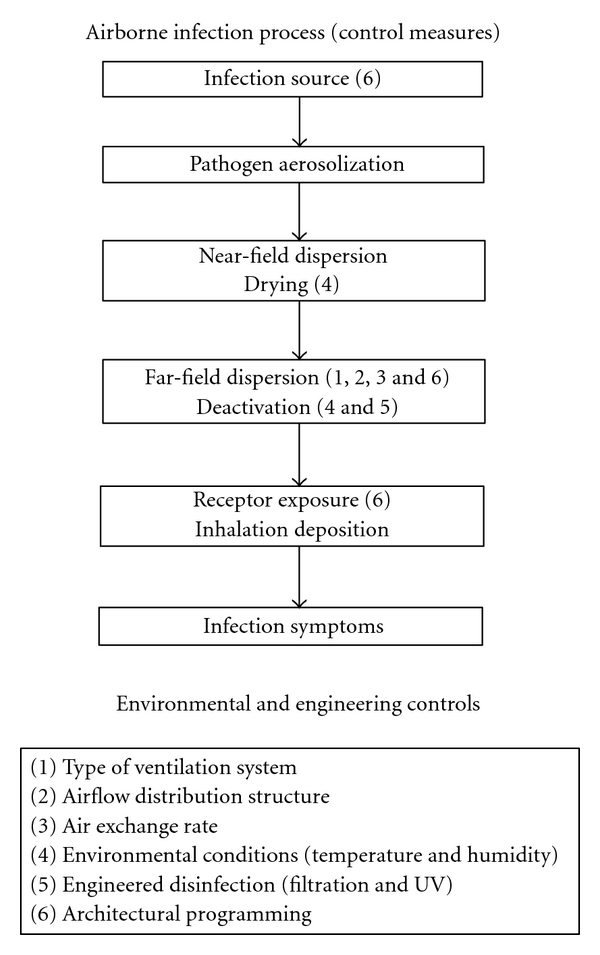
Airborne infection process and influential environmental/engineering controls.

Figure 1 shows the airborne infection pathway and the environmental and engineering controls that may influence the steps along the path. In Section 2, we review each step of the infection pathway. As will be indicated therein, each step has been the subject of earlier reviews, but our focus is on factors that can influence the relative risks of different ventilation systems. In Section 3, we discuss the engineering controls that can influence the infection steps. Also discussed are the relative merits of different ventilation systems (with a focus on health care facilities) and whether or not the models of Section 2 may be applied for risk estimation.  Section 4 concludes with a discussion of the challenges remaining for methods that can be used for health care facility ventilation design.

## 2. Predicting Airborne Infection Risk: From Source to Receptor

For effective ventilation design of a health care facility, one needs to be able to quantify and predict airborne infection risk. The informed selection of one ventilation design strategy over another requires the use of suitable metrics. To provide a useful prediction, many input parameters need to be supplied to an airborne infection risk model or experiment. The accuracy and extent of these parameters, of course, depend on the model or experiment complexity and the desired level of detail for the expected results. The key factors of the airborne infection process, which determine the organization of our discussion, are present in the Wells-Riley risk model for a well-mixed room [9]

(1) $$\begin{array}{c} P_{I}=\frac{C}{S}=1-\exp\left(\frac{Iqpt}{Q}\right), \end{array}$$

where *P*_*I*_ is the probability of infection, *C* is the number of infection cases, *S* is the number of susceptible persons, *I* is the number of infectors, *q* is the quanta generation rate, *p* is the pulmonary ventilation rate of a person (inhalation), *t* is the exposure time interval, and *Q* is the room ventilation rate with clean air. As implied by this equation, one needs to know *I*, *q*, *p*, *t*, and *Q* in order to quantify infection risk.

This model is useful but only for the simple case of a well-mixed room, where airborne pathogens are randomly distributed in space. More parameters and complications arise for scenarios in which the air is not well-mixed. In addition, empirical data need to exist for *q* that quantifies a minimum dose of pathogens that has been observed to infect a person. In Section 2.5, we will consider and compare more sophisticated risk models, but they all involve the same factors: aerosol generation, pathogen transport, infectivity loss, and inhalation and deposition.

### 2.1. Generation of Aerosols

#### 2.1.1. Categories of Airborne Aerosols

Aerosols are suspensions of fine solid or liquid particles in a gas. The medical profession reserves the term *airborne* for aerosols that are transported by air currents over long time periods (minutes) and large distances (>1 m). Thus, small aerosols contribute to the *airborne infection mode,* while larger aerosols (which settle out quickly) contribute to the *droplet infection mode*. These are some variations in how the terms are used in the literature [5, 6].

There is agreement that aerosols smaller than 5 *μ*m in aerodynamic diameter (also called droplet nuclei [5]) contribute to airborne infection [1, 6]. However, Tellier [1] considers aerosols larger than 20 *μ*m, while Tang et al. [10] consider aerosols larger than 60 *μ*m as contributing to droplet infection. Some authors also define an intermediate size range where aerosols contribute to infection via both airborne and droplet modes. This intermediate behavior depends on particular geometrical settings, airflow patterns in ventilation, and also aerosol response to the surrounding environment [1, 10].

Particular care must be given to aerosols that change in size during the time of flight due to evaporation. An aerosol may move from the droplet regime towards the airborne regime due to mass loss. Aerosol composition and environmental factors such as temperature and relative humidity determine such changes and must carefully be considered in any study [1, 6, 7, 10, 11].

There are hundreds of airborne communicable pathogens [6, 10, 12] falling into three major categories: viruses, bacteria, and fungal spores. Viruses are the smallest with diameters of 0.02–0.3 *μ*m. Bacteria have diameters in the range of 0.5–10 *μ*m. Spores are the largest with diameters in the range of 0.5–30 *μ*m [12].

Human activities are key sources for dispersal of airborne pathogens. These include respiratory activities (breathing, speaking, coughing, sneezing, etc.), showering, flushing, using tap water (atomization of infectious aerosols, particularly bacteria present in the water or in the local plumbing), sewage aerosolization from toilets and its transport in building down-pipe systems, and wet-cleaning of indoor surfaces [7]. Other human activities such as bed making, walking on carpet, or skin shedding, cause resuspension of aerosols from surfaces [8].

In addition, various medical procedures also contribute to pathogen transmission. Some procedures that may increase droplet nuclei generation are intubation, cardiopulmonary resuscitation, bronchoscopy, autopsy, and surgery with high-speed devices. Presently, there is no precise list of such procedures, and neither has there been any study on the impact of ventilation design on the spread of pathogens released by high-risk procedures [5].

Aside from these sources, each building facility has its own microbial ecology that supports the growth of certain kinds of pathogens and suppresses the growth of others. For example, heating ventilation and air conditioning (HVAC) system components such as filters, cooling coils, air intakes, and porous insulation in air ducts can support the growth and dissemination of spores in certain areas. On the other hand, sufficient sunlight and natural ventilation in other areas may disinfect pathogens [11, 12].

#### 2.1.2. Expiratory Aerosols

Expiratory droplets are particularly important in the spread of airborne infection. Human expirations (breathing, coughing, and sneezing) create the smallest aerosols compared to other sources. Particular attention is paid to human expiratory sources of aerosols for the remainder of this paper.

Coughs and sneezes were studied by Jennison [13] who applied high-speed photography to track the size and motion of droplets as subjects sneezed. Seventy years ago, it was not possible to track aerosols smaller than 100 *μ*m. Nevertheless, Jennison determined the important length and time scales of sneezes.

Duguid [14] studied the sizes of droplets produced by sneezing, coughing, and speaking using microscopic measurement of stain marks found on slides exposed directly to air exhaled from the mouth. He was able to detect droplets sized in the range of 1–2000 *μ*m. Fairchild and Stamper [15] measured droplets in exhaled breath using an optical particle counter (OPC) in the range of 0.09–3.0 *μ*m. Papineni and Rosenthal [16] studied the size distribution of droplets exhaled by healthy individuals while mouth breathing, nose breathing, talking, and coughing. They used an OPC and an analytical transmission electron microscope (ATEM). The OPC indicated that the majority of droplets were under 1 *μ*m. ATEM measurements were conducted by collecting droplets on slides and viewing their size under microscope after evaporation. The original droplet size was corrected with a calculation. They confirmed the existence of larger droplets in exhaled breath as opposed to nose breathing. Yang et al. [17] studied the size distribution of droplets experimentally using the aerodynamic particle spectrometer (APS) and the scanning mobility particle spectrometer (SMPS). Their samples were bagged before analysis; hence, significant evaporation and droplet settling may have occurred. An experimental study by Chao et al. [18] considered characteristics of a real cough just after the mouth opening using interferometric Mie imaging (IMI). They found that droplets are in the range of 2–2000 *μ*m (corresponding to the entire measurement range of IMI).

The large variation in reported droplet size can be attributed to three major causes: (i) the sensitivity of different measurement techniques, (ii) the unrepeatable nature of coughs and sneezes for each subject as well as the variability of coughs and sneezes among different subjects, and (iii) the evaporation of droplets at different time scales according to their initial size. Size distribution data found in the literature are summarized in Table 1.

The physiology of coughing is described by McCool [20] as a three-phase reflex: inspiration, compression, and expiration. The peak flow rate in a cough may reach as high as 12 L/s. Piirilä and Sovijarvi [21] performed an objective assessment of coughing. They investigated the cough as a primitive reflex typically consisting of an initiating deep inspiration, glottal closure, and an explosive expiration accompanied by a sound. The flow characteristics of a cough were reported to vary from person to person. They reported that the durations of the different phases of the cough reflex can be easily measured on a graph of flow versus time. They suggested that the duration of the glottal closure during the compressive phase of cough varies in the range of 0.09–1.01 s. They also defined a useful parameter in characterizing the cough, the cough peak expiratory flow rate (CPEF). Nishino [22] explains the physiology of coughing and sneezing in detail and points out the similarities and differences between the two. The flow dynamics of a sneeze are similar to the cough in time variation of flow rate. However, the peak velocities are higher, and in addition to mouth exhalation, a small fraction of the exhalation exits the nose. For sneezes, Jennison [13] reported exit velocities as high as 90 m/s with peak velocity time (PVT) of 57 ms. The total sneeze time was reported in the range of 0.07–0.20 s. Zhu et al. [23] performed particle image velocimetry (PIV) measurements and computational fluid dynamics (CFD) simulations of cough droplet dispersion in a calm background. Experimentally, they found that the initial velocity of coughs varies in the range of 6–22 m/s and the amount of saliva injected is in the range of 6.1–7.7 mg. Chao et al. [18] reported an average expiration air velocity of 11.7 m/s for coughing and 3.9 m/s for speaking.

Gupta et al. [24] performed an experimental study to characterize the flow rate versus time profile of a human exhalation. They have combined gamma-probability-distribution functions to fit experimental data. Such functions will be particularly useful for setting cough and sneeze boundary conditions for CFD studies. They characterize the complete distribution by only three parameters: cough peak flow rate (CPFR), peak velocity time (PVT), and cough expired volume (CEV). These boundary conditions were implemented in a CFD simulation by Aliabadi et al. [25]. They demonstrated that volatile cough and sneeze aerosols evaporate at different time scales according to their size. In general, small droplets (<20 *μ*m) evaporate at much faster time scales (milliseconds) than larger droplets (>50 *μ*m) for which the evaporation time is in the order of seconds. The most important factors in evaporation rate are temperature and relative humidity in the ambient air.

Höppe [26] pioneered the measurement of expiration temperatures in different climatic conditions. He studied the nasal and oral exhalation temperatures as a function of environment temperature (5°C –33°C) and environment relative humidities (10%–90%). Noticeable variabilities in exhalation temperatures were observed. Similarly, McFadden et al. [27] provided thermal mapping of the human airways using measurements by inserting fine thermistor probes into the respiratory tract. They found that at normal-to-high rate breathing, the temperature in the upper airway system is in the range of 33.9°C –35.5°C.

### 2.2. Dispersion, Heat, and Mass Transfer

After aerosol generation, the next step in the infection pathway is the dispersion of airborne pathogens in ventilation space, possibly towards potential suspects. This dispersion is a function of many variables such as aerosol size, mean and fluctuating velocities of air, temperature, and the rate at which the aerosol is transferring mass or heat with the environment (i.e., evaporation or cooling/heating). These processes cannot be modeled analytically except in the most idealized cases. Rather, CFD is required to model both the continuous phase (the air) and the discrete phase (the aerosols).

#### 2.2.1. Modeling Airflow

Solving the continuum phase (air) in ventilation flow requires the integration and solution of mass, momentum, and energy equations, normally using finite volume discretization methods [28].

The fluid flow regime is determined largely by the Reynolds number (dimensionless ratio of intertial to viscous forces, *Re* = *VL*/*ν*) and Grashof number (dimensionless ratio of the buoyancy to viscous forces, Gr = *gβ*(*T*_*s*_ − *T*_*∞*_)*L* ^3^/*ν* ^2^). In these equations, *V* is velocity, *L* is length scale, *ν* is kinematic viscosity, *g* is gravitational acceleration, *β* is coefficient of thermal expansion, *T*_*s*_ is surface temperature, and *T*_*∞*_ is far-field temperature. Depending on the room geometry, transition from laminar to turbulent flow occurs at *Re* ~ *O*(10^3^), and buoyancy-driven flows (e.g., thermal plumes) become important for Gr/Re^2^ > *O*(10). The process of airborne infection in a room involves widely differing scales. For example, the flow in the vicinity of a sneeze is highly turbulent and not strongly influenced by gravity or buoyancy. In contrast, over longer times (minutes) and larger length scales (full room), the turbulence intensity is less and the influence of gravity or buoyancy may be larger. The heat and mass transfer to an expiratory droplet is determined by flow conditions in the immediate distance (1–100 *μ*m) around the droplet, which is always laminar due to the small relevant length scales and the small aerosol-air relative velocity.

Typically, some form of turbulence modelling is needed for room-scale simulations, yet modeling turbulence accurately is the limiting factor for continuum phase modeling for two reasons: (i) the physics of turbulence is not well understood and (ii) accurate modeling of turbulence is computationally very expensive.

The most accurate way to model turbulence is direct numerical simulation (DNS). In this technique, the eddies (fluid structures) of all length scales (from small to large) are resolved. This technique, however, demands immense computational power with increasing *Re* or Gr numbers, and, hence, is not applied in ventilation simulations.

As a compromise, the large eddy simulation (LES) technique has been developed that resolves larger eddies but uses simple models of the smaller scales of the flow. The basic motivation behind this idea is that large eddies are the primary mechanisms transporting aerosols over large distances. This reduces the computation cost substantially, but it still poses challenges for modeling ventilation airflow: (i) the required computation cost is still high, (ii) many realizations of the airflow are necessary for statistically significant results, and (iii) original perturbation fields for the flow are not known or are difficult to generate [29].

A less computationally costly approach in modeling turbulence is Reynolds averaged Navier-Stokes (RANS) modeling. This approach does not resolve any real-time scales of the flow but instead considers time-averaged and fluctuating components of the flow separately. These models report time-averaged parameters of turbulence such as *kinetic energy* and *dissipation rate*. Many variations of RANS models are available (*k* − *ϵ*, *k* − *ω*, *v* ^2^ *f*, Reynolds stress model (RSM), etc.). Many researchers have used the standard or realizable *k* − *ϵ* turbulence model in solving ventilation airflow [30–32]. Other researchers have predicted ventilation airflow using renormalization group (RNG) *k* − *ϵ* turbulence model. Compared to the standard and realizable *k* − *ϵ* models, the RNG model has a better ability to model both high and low *Re* or Gr numbers in the same flow [29, 32–38]. Most RANS models are computationally economic and provide useful results, particularly when *qualitative* results are sought. However, they do not consider the anisotropy of the turbulence and often have difficulty to reach a converged solution. One remedy is to use the Reynolds stress model (RSM) that allows for anisotropy of turbulence and provides better results than other RANS models if the initial solution is guessed properly.

An alternative approach is to combine RANS and LES to obtain a detached eddy simulation (DES) in which LES is used in areas of strong large-scale unsteadiness such as in the wake of a person, while RANS is used to model the flow elsewhere. In this technique, LES is used where the grid is sufficiently fine so that large eddies can be resolved accurately [29].

A summary of the advantages and disadvantages of the major turbulence models is provided in Table 2. Due to its relative computational speed, RANS is the only approach used today in the engineering design of ventilation systems.

#### 2.2.2. Modeling Aerosol Dispersion, Heat, and Mass Transfer

Particle dispersion can be modeled using several approaches. The simplest approach is to assume that aerosols behave like gases (true only for submicrometer aerosols) and to solve for gas concentration transport in the conservation equations. Many studies have used this approach [32, 33, 35, 36], but it cannot be used to predict the transfer of heat and mass with the continuum phase. Also, aerosols larger than 1 *μ*m are affected by other dispersion forces including gravity, which are not accounted for in gas dispersion modeling.

Alternatively, the trajectory of an aerosol can be determined by the force balance that equates the aerosol inertia with the forces acting on it [25, 39]

(2) $$\begin{array}{c} \frac{d{\vec{u}}_{p}}{dt}=F_{D}\left(\vec{u}-{\vec{u}}_{p}\right)+\frac{\vec{g}\left(\rho_{p}-\rho \right)}{\rho_{p}}+\vec{F}, \end{array}$$

where ${\vec{u}}_{p}$ is the aerosol velocity, $\vec{u}$ is the continuum phase velocity, *F*_*D*_ is drag acceleration per unit velocity (determined by Stokes law for the smallest aerosols or empirical drag coefficients for larger aerosols), *g* is gravitational acceleration, *ρ*_*p*_ is aerosol density, *ρ* is continuum phase density, and $\vec{F}$ is the acceleration per unit mass caused by the Brownian force.

Neglecting radiation, the mechanisms of aerosol mass, and temperature change are convection and evaporation. Having the time rate of change of aerosol mass and the convective heat transfer coefficient, the energy balance equation for an aerosol may be written as

(3) $$\begin{array}{c} m_{p}c_{p}\frac{dT_{p}}{dt}=hA_{p}\left(T_{\infty }-T_{p}\right)+\frac{dm_{p}}{dt}h_{fg}, \end{array}$$

where *m*_*p*_ is aerosol mass, *c*_*p*_ is aerosol specific heat capacity, *T*_*p*_ is aerosol temperature, *h* is convective heat transfer coefficient, *A*_*p*_ is aerosol surface area, *T*_*∞*_ is the far-field continuum phase temperature, and *h*_*fg*_ is the latent heat of vaporization.

To produce statistically significant results, a large ensemble of droplets of various sizes are tracked stochastically, and bin-based mean dispersion locations and diameters are reported for a distribution of aerosols [40]. Many literature studies adopt this modeling approach [30–32, 34, 38].

### 2.3. Viability and Infectivity

The term *viability* refers to the survival of pathogens in a given set of environmental conditions. Pathogens are termed *infective* only if they are able to attack host cells and reproduce themselves [41]. Thus, all infective pathogens are also viable, but the converse is not necessarily true [42, 43]. This paper does not focus on detailed and complex mechanisms of infection; however, a cursory review is needed here, because the uncertainty in infectivity data can dominate risk estimates and strongly influence ventilation design.

During aerosolization, fluid shear stresses can inactivate some pathogens. Furthermore, following aerosolization, the viability of a pathogen changes as a function of various environmental conditions, including the relative humidity, temperature, oxygen and ozone concentration, open air factor (OAF), and electromagnetic radiation [43]. On the other hand, the infectious disease process in a host depends on the pathogen concentration (infection dose) and virulence (disease promoting factors) that enable an agent to overcome the physical and immunologic defense mechanisms in the host [11].

It is important to note that innate and adaptive host immune responses (e.g., past exposures and/or vaccination) will modify the response to any exposure considerably. The following responses may be possible: (i) exposed but not infected, (ii) exposed and infected but not diseased (due to rapid immune clearance primed by past exposures and/or vaccination, (iii) exposed, infected, and diseased. In addition, infectivity of a virus depends on previous infection of a host with another disease. Hall et al. [44] studied viral shedding patterns of ambulatory children with influenza B. They found that the infection symptoms varied in type and time depending on previous infections/diseases that the children already had. These intrahost mechanisms/factors are not within the scope of this paper.

#### 2.3.1. Viability and Infectivity Measurements

Numerous techniques have been used to measure the viability and infectivity of airborne pathogens. Four major classes of techniques are reviewed below.

*Animal tests* for airborne infection consider many physical and biological aspects of pathogen viability and infectivity. Some researchers have reported studies using guinea pigs, monkeys, and cattle [1, 42, 45]. Although many pathogenic species are common to humans and these animals, some difficulties exist in extrapolating the viability and infectivity measurements to humans using these tests. The respiratory tracts of humans and animals have different physiologies. As a result, the respiratory tract size-dependent filtration and deposition efficiencies vary greatly from one creature to another [42]. In addition, the true infectivity of airborne pathogens is a function of *both* the source and the receptor. The defense mechanisms in humans and other creatures are different, resulting in different infectivities for a given pathogen.

A large class of methods are termed *culture methods*, since they are based on the principle of cell growth. These methods are among the most popular and classical techniques used to measure viability and infectivity of airborne pathogens. Using these methods, a sample of airborne pathogens is collected on a media (e.g., agar plates) and incubated over time in favorable conditions (temperature, relative humidity, and chemical composition) to investigate how the pathogens multiply. The colony-forming unit (CFU) will then be the measure of the pathogen's ability to reproduce [46, 47].

 It is important to account for viability and physical losses of pathogenic aerosols separately [48]. To achieve this, some researchers have added aerostable spores or radioactively marked cells of a known proportion to the pathogen of interest whose viability is going to be studied. The viability for these aerostable spores or radioactive marked cells does not change in a wide range of environmental conditions [48–51]. Some difficulties exist with traditional culture methods. The capturing of very fine pathogen-containing aerosols on solid media (e.g., agar plates) or liquid media (e.g., all-glass impingers) may be difficult [41]. In addition, viable and reproducible cells may be collected in agglomerates whose CFU will underestimate the actual count.

Another large class of methods are termed *molecular methods*. These methods do not depend on cell growth and can detect both reproducible and nonreproducible pathogens [41]. The reverse transcriptase-polymerase chain reaction (RT-PCR) technique permits detection of a single-pathogen DNA or RNA by making a billion-fold copies of it [1, 7]. Although accurate in detecting the genomes (DNA or RNA), an important limitation of this technique is that RT-PCR cannot establish the infectivity of the viral aerosols detected [1]. Some researchers have used direct microscopy to provide a total count of viable pathogens in a prepared solution. One such technique relies on color staining of pathogens in the solution by adding chemicals (e.g., acids) to which pathogens respond [41].

Yet another large class of methods are termed *plaque assay methods*. The main characteristic of these methods is that the activity of the species of interest is observed in an organism or organic sample. For infectivity tests, the ability of a pathogen to attack and damage a cell is measured. To form a plaque assay, aerosols are sampled (e.g., with all-glass impingers) and multifold dilutions of a pathogen stock are prepared. Then, standard volume aliquots are inoculated and incubated in the vicinity of susceptible cell samples on plates. A plaque forms around damaged cells, and it grows until limited by the gel structure of the plate. This visual plaque allows for titer calculation of the pathogen in plaque-forming units (PFUs) per unit space. Sometimes, living cells are dyed such that the color contrast between the plaque and living cells are pronounced [52–54]. Plaque assay methods reveal information about the ability of the pathogen to attach and infect living cells under favorable conditions. However, it is very difficult to extrapolate true infectivity of a pathogen for the host due to the variability of host factors already listed.  Table 3 provides a summary of viability and infectivity measurement techniques for airborne pathogens.

The true viability and infectivity of airborne pathogens depend on complex physical and biological mechanisms that affect the survival of pathogens while suspended in air, their deposition onto susceptible sites in the host, and their ability to defeat the defense mechanisms of the host. None of the existing measurement techniques accurately accounts for *all* of these mechanisms. As a result, it must be understood that any measurement technique, at best, approximates true viability and infectivity focusing on only limited aspects of viability or infectivity. For example, if molecular methods are used, accurate counts of pathogens are possible, but the estimates for their reproducibility and true interaction with the host are compromised. On the other hand, if plaque assay methods are used, some degree of pathogen interaction with the host is accounted for, while an accurate count of pathogens is compromised. Hence, the validity for prediction of airborne infection risk in a given building ventilation setting is limited to the type of viability and infectivity measurement technique used.

#### 2.3.2. Environmental Factors Affecting Infectivity and Viability

Many environmental stressors are responsible for the loss of viability and infectivity in aerosolized pathogens.  Table 4 shows the stresses and the target cell components in order of significance [43].

Upon aerosolization, bacteria and viruses desiccate when dispersed in liquid suspensions such as saliva and then surrounded by relatively dry air. Loss of water is the greatest environmental stressor to pathogens and results in a loss of viability. On the other hand, the high relative humidity level in the respiratory tract promotes aerosol growth and affects the deposition site and efficiency as well as some repair mechanisms in the viability of microbes upon inhalation.

The relative simplicity of viral structure explains why the results of aerosol inactivation studies are more consistent for viruses than for bacteria. Inactivation of viruses is affected by the following variables: (i) viruses with lipids in their outer membrane are more stable at low relative humidities (20%–30%) than at high relative humidities, (ii) viruses without lipids are more stable at high relative humidities (70%–90%) than at low relative humidities, (iii) the nucleic acid for viruses without lipid membrane may be isolated and not detected during desiccation, while it can be recovered by prehumidification at sampling, (iv) minimal survival for both lipid and nonlipid membrane viruses occurs at intermediate relative humidities (40%–70%) [55, 56]. Example viruses with lipid membranes include Langat, Semliki forest, Vesicular Stomatitis, Vaccinia, and influenza [43]. Some nonlipid membrane viruses include respiratory Adenoviruses and Rhinoviruses [56].

Due to the greater complexity of their biochemistry, structure, and organization, it is difficult to generalize the effect of relative humidity on bacterial viability. Gram-negative bacteria (bacteria that do not retain crystal violet dye in the Gram-staining protocol) such as *Serratia marcescens*, *Escherichia coli*, *Salmonella pullorum*, *Salmonella derby*, *Pseudomonas aeruginosa*, and *Proteus vulgaris* have lower viability at intermediate (50%–70%) to high (70%–90%) relative humidity environments. Also, some Gram-positive bacteria (bacteria that are stained dark blue or violet by the Gram staining protocol) such as *Staphylococcus albus*, *Streptococcus haemolyticus*, *Bacillus subtilis*, and *Streptococcus pneumoniae* (type 1) are found to have lower viability at intermediate relative humidities. In contrast, Gram-negative Klebsiella pneumoniae demonstrates relative stability (higher viability) at intermediate relative humidities. Some studies have also shown that Gram-negative Pasteurella species survive better at high relative humidities [56].

Aside from whether the bacteria are Gram-positive or Gram-negative, whether the bacteria are *dry-disseminated* or *wet-disseminated* also affects viability. Cox [43] has defined the former as meaning that the organism is aerosolized from a dry dust or freeze-dried powder, and the latter means that the organism is aerosolized from a liquid suspension, for example, human mucus or saliva. Cox [43] has found that dry-disseminated bacteria absorb water from the environment, while the wet-disseminated bacteria lose water to the environment by evaporation. Such changes in water content (i.e., rehydration or desiccation) affect the viability behavior. For example, Cox [43] found that for wet dissemination of *Pasteurella*, viability reaches a minimum at 50%–55% relative humidity, while for dry dissemination it reaches a minimum at 75% relative humidity.

Fungi have been less studied under laboratory conditions and most experimental data have been obtained by monitoring fungal levels in indoor and outdoor environments. Fungi are expected to be present at higher levels in naturally ventilated buildings. Generally, higher relative humidities support the survival of fungi [56].

The vapor pressure, and therefore the relative humidity, is dependent on temperature. As a result, it is difficult to completely separate the effects of humidity from temperature. However, studies that do attempt to find the effect of temperature on aerosolized pathogen stability have generally shown a decrease in viability when temperature increases [55].

Temperature can affect the state of viral proteins (including enzymes) and the virus genome (RNA or DNA). DNA viruses are generally more stable than RNA. Usually, an increase in the temperature results in a decrease in virus viability. Maintaining temperatures above 60°C for 60 min is generally enough to inactivate most viruses. The presence of surrounding organic material (e.g., blood, saliva, mucus, etc.) can protect viruses against temperature stresses [56].

Viral culture experiments show that temperatures as low as 7°C -8°C are optimal for airborne influenza survival, with survival decreasing progressively at moderate temperatures of 20.5°C–24°C. This relationship holds true for a range of relative humidities (23%–81%). Other *in vivo* experiments with guinea pigs confirm that influenza transmits through air better in cold and dry conditions. Recent experiments have shown that higher temperatures of about 30°C actually block the aerosol transmission of influenza [56].

Studies generally show that at temperature above about 24°C, bacteria appear to universally lose viability. This reduced viability has been observed in members of Gram-positive, Gram-negative, and intracellular bacteria: *Pseudomonas*, *Pasteurella*, *Salmonella*, *Serratia*, *Escherichia*, *Bacillus*, *Brodetella*, *Chlamydia*, and *Mycoplasma* species [56].

Generally, higher temperatures support fungi survival. The indoor and outdoor concentration of *Aspergillus* and *Penicillium* species may vary considerably in both winter and summer, as well as in urban or suburban environments, with higher temperature and relative humidity with suburban areas being generally more favorable for higher airborne spore concentrations [56].  Table 5 shows a summary of the effect of temperature and relative humidity on the survival of airborne pathogens.

Comparing the survival of pathogens in the laboratory with those outdoors shows that under the same conditions of photoactivity, relative humidity, and temperature, outdoor air is often more toxic to pathogens than indoor air, especially in urban areas [43, 57]. Cox [57] attributes this inactivation to an open air factor (OAF). OAF inactivation is probably caused by a multitude of factors including pollutant concentration, relative humidity, pressure fluctuations, and air ions [43].

Although the exact nature of the (OAF) is not known, various experimental efforts have been undertaken to correlate OAF mechanisms with known mechanisms of pathogen inactivation. In a study, various concentrations of O_3_, NO, NO_2_, SO_2_, C_3_H_8_, C_3_H_6_, C_2_H_4_, and C_2_H_2_ have been introduced to inactivate pathogens. In separate experiments, various exposures of OAF have been introduced to inactivate organisms. It was found that OAF is most closely correlated with ozone (O_3_) and C_3_H_6_ [57].

Aerosol inactivation caused by electromagnetic radiation is observed to be wavelength dependent. Also, relative humidity, oxygen concentration, aerosol age, and the presence of other gases affect the electromagnetic radiation damage to viability. Shorter and more energetic wavelengths (X-rays and gamma rays) can break the DNA of pathogens. UV radiation acts as an energy source for the production of thymidine dimers. Longer and visible wavelengths are shown to affect cytochromes in the mitochondria of yeasts and bacteria. Another study also shows that survival of aerosolized bacteria around sewage treatment plants was higher at night compared to daytime [55].

#### 2.3.3. Viability and Infectivity Models

Viability and infectivity are often difficult to separate, so it is common to model their product as a single parameter (equivalent to assuming that all viable organisms are infective). Inactivation of microbial aerosols is a function of many parameters: temperature, suspension fluid chemistry, relative humidity, oxygen, and time. However, integration of all of these factors in a model is a complicated task, because the exact inactivation mechanisms for many microbes are not well understood. In addition, many factors have synergistic effects (e.g., temperature and relative humidity), making it difficult to formulate a comprehensive model. Finally, the response to environmental stressors is unique to each organism (e.g., genetic predisposition). Thus, most developed models in the literature are empirical, only considering a few of these factors, usually time and another factor like temperature or relative humidity. The model parameters are fit experimentally for the viability decay of each microbial aerosol of interest.

During and after the aerosolization of a microbial solution, there is a period of stabilization. During this initial time period, many microbes experience shear stresses and disintegrate. Also, aerosols of interest that remain airborne experience rapid evaporation (during the first 10 s) with temperature, relative humidity, and concentration of certain solutes in the droplet varying rapidly to a level that may be toxic to the microbes. The initial stabilization period is fast relative to the airborne lifetime of aerosols. Also, the interplay of various environmental stressors are far too complicated to be understood and modeled with the current methodologies [58].

Exponential decay is often used to model viability; although a gross simplification, it often performs as well as detailed models with 20 or more parameters [55]. For any set of environmental conditions, the exponential decay model is given by

(4) $$\begin{array}{c} V_{t}=V_{0}e^{-kt}, \end{array}$$

where *V*_0_ is % viability at time zero, *V*_*t*_ is viability at time *t*, and *k* is decay constant. Many studies have used the standard exponential decay model to fit curves to viability data or predict viability in some other modeling context [12, 54, 59]. Some researchers have extended the standard exponential model by expressing the decay parameter as a variable governed by water activity and critical water activity in the suspension solution. Posada et al. [58] fit other constants to obtain an exponential expression for the decay variable.

Although the exponential decay model (4) offers many advantages and it is easy to use, it has one major drawback. It predicts the viability to be near zero when the aerosol age is large. This is contrary to experimental data that show an initial fast decay followed by a slow decay causing viability to asymptotically approach a nonzero minimum value [57]. As a result, particularly when using the exponential decay model for airborne infection risk prediction over long periods, extreme care must be taken not to underestimate the risk.

To overcome this difficulty with the exponential decay model, a series of higher-order kinetic models have been developed by Cox [57]. As explained, each model considers only up to two parameters, one of which is time and the other the relative humidity, temperature, or oxygen. As described before, relative humidity has the greatest impact on microbial survival. To use kinetic models, one needs to have experimental data for a given set of relative humidities, temperature, or oxygen for a particular pathogen. One then fits the data with a few constants to obtain the model.

The other alternative to the exponential model (4) is the catastrophe model. In classical treatments, chemical reactions are assumed to proceed continuously, whereas close examination suggests this is only an approximation, because at the molecular level, individual reactions are not continuous events. The continuum approximation becomes more accurate as the number of molecules becomes large. Loss of viability in a small aerosol has a discontinuous nature, since only a small quantity of microbes are concerned. A microbe is either alive or dead, and the sudden change between these two states is termed catastrophe [57]. The mathematical model of catastrophe theory involves describing the potential energy of the system in terms of control parameters. For some range of values for the control parameters, the potential energy curve has a stable equilibrium, which represents the viable state. If the control parameters are changed, there may result a catastrophic drop in potential energy, which leads to the inactivated or nonequilibrium state [57].

 High-order kinetic and catastrophe models for pathogen inactivation are more biologically plausible than the exponential model, but seldom is there sufficient data to support the more sophisticated models. The advantages and disadvantages of the viability models described above are listed in Table 6.

### 2.4. Inhalation and Deposition

#### 2.4.1. Respiratory System Construction

The human respiratory system is comprised of three regions: (i) an upper respiratory portion consisting of the nasopharynx and mouth, (ii) conducting air passages of the larynx, trachea, and large bronchi, and (iii) a respiratory gaseous exchange region formed by secondary bronchi, terminal bronchioles, and alveoli [43]. Cells lining these areas have different functions, with ciliated, mucous producing cells in the nasopharynx, descending to single cells in contact with interstitial fluid forming the alveoli.

#### 2.4.2. Deposition Mechanisms

Deposition of aerosols in the respiratory tract occurs via different physical mechanisms. Aerosols smaller than 0.1 *μ*m in diameter are transported onto human airway surfaces by Brownian diffusion. For aerosols roughly between 0.1 *μ*m and 1.0 *μ*m, deposition may occur due to the combined action of Brownian diffusion and impaction. For aerosols increasing in size from 1 *μ*m to about 1000 *μ*m, the deposition mechanism shifts from impaction to sedimentation (i.e., gravitational settling) [55].

Deposition of aerosols in the respiratory tract depends on the tract morphology. In addition, both the respiration mode and breathing pattern must be considered in modeling aerosol deposition in the human lung. Humans have the ability to breathe through either the nose or the mouth. The breathing pattern can occur either as regulated or spontaneous breathing. The breathing pattern is often described in terms of tidal volume and flow rate. In general, larger tidal volumes result in higher aerosol deposition in the lung as aerosol-laden air penetrates deeper into the lung. Lower flow rates also result in higher aerosol deposition by sedimentation and diffusion processes [60, 61].

Temperature and relative humidity in the respiratory tract vary with type of respiration, and anatomical location. Generally, a temperature of 37°C and a relative humidity of 99.5% may be assumed for nasal respiration. For oral respiration a temperature of 37°C and a relative humidity of 90% may be assumed. The relative humidity can be assumed to increase 1% per airway generation (branching) until a maximum of 99.5% is reached. Relative humidity and temperature affect the growth of hygroscopic aerosols in the human lung. This causes the aerosol diameter and density to change. As a result, actual aerosol sizes for *in vivo* and *in vitro* experiments may be different [60].

#### 2.4.3. Respiratory Tract Deposition Models

Aerosol deposition in the lungs has been modeled both empirically and mechanistically. In empirical models, the fluid and aerosol dynamics associated with respiration are incorporated by simplified expressions [60]. Mechanistic modeling of the deposition of aerosols in the respiratory tract requires the description of the morphology of the airways. Both the overall branching structure of the airway tree and dimensions (e.g., diameters and lengths) of each airway must be considered. Both idealized morphology models and models based on specific experimental observations have been used in aerosol deposition modeling [60].

### 2.5. Summary: Infection Risk Models

The physical and biological processes reviewed up to this section determine (explicitly or implicitly) the airborne transmissibility of disease from the source to receptor. A model of this transmission process is referred to here as an *infection risk model*.

Infection risk models can be either *deterministic* or *stochastic*. In deterministic models, each individual is hypothesized to have an inherent tolerance dose for an infectious agent. When he or she intakes a dose higher than this tolerance, infection occurs. Otherwise, it does not. On the other hand, stochastic models do not determine whether an individual will fall sick under certain dosage conditions. Instead, the model estimates a probability of acquiring the infection under the intake dosage [62, 63]. The distinction between stochastic and deterministic models is more philosophical than practical, because even if an underlying infection process is deterministic, there are always a host of unresolved parameters (ranging from genetic variations of host and pathogen to the turbulent transport from source to receptor) such that all practical models reduce to a probabilistic calculation.

A further categorization of infection risk models is *threshold* versus *nonthreshold*. Threshold models assume that a minimum number of pathogens are necessary to infect a subject, whereas nonthreshold models assume that any number of pathogens, in principle, can cause infection [62].

#### 2.5.1. Wells-Riley Infection Model

Riley et al. [9] developed the first airborne infection model in an epidemiological study in a measles outbreak. Their formulation (1) is based on the concept of *quanta of infection*. This quantum is defined as the number of infectious airborne aerosols required to infect a person. The Wells-Riley equation assumes a *well-mixed* room air and a steady-state infectious aerosol concentration which varies with the ventilation rate (*Q*). The biological decay of the airborne pathogens are not explicitly considered in this equation; however, this information is implicitly embedded in the model by the quantum generation (*q*). Since Wells-Riley model is used with experimental measurements of the quanta of infection, it considers many implicit complexities.

Various researchers have used the *well-mixed* Wells-Riley model to predict infection risk [12, 64]. To improve the model, other studies have incorporated effects of respirator filtration, viability loss of the pathogens, deposition loss of infectious pathogens, and inactivation of pathogens by ultraviolet irradiation [62, 65]. Although these efforts have come a long way, they do not provide details of spatial distribution of risk in a given space. To overcome these difficulties, Qian et al. [33] have integrated the Wells-Riley equation into a CFD model to predict the spatial distribution of risk in a ventilated space. Essentially, the well-mixed model is applied to small subdomains of the room.

#### 2.5.2. Dose-Response Infection Model

Dose-response models require infection dose data to construct the dose-response relationship. This data is obtained experimentally in such a way that a large susceptible population is exposed to different doses of a pathogen, and it is observed what fraction of the population develop an infection. For example, the dosage that causes 50% of the population to fall sick is called ID_50_.

The tolerance dose concept is biologically plausible, since it considers the variation of immune status and the host's sensitivity in the subjects. There are two opposing views of the process of microbial infection of a host in so far as derivation of dose-response relationship is concerned. The first model can be described as the deterministic hypothesis, which assumes that complete cooperation among pathogens to cause infection in the host. Under this hypothesis, each host organism is assumed to have an inherent minimal infective dose, and if it is exposed to a dose in excess of this minimal amount, then an observed response will result. The second model can be described as stochastic hypothesis, which assumes pathogens work independently and each of them can potentially cause infection in the host (single hit) [63].

Deterministic dose-response models are direct implementations of the tolerance dose concept. These models require experimental infection data for a population to fit a curve for the distribution of tolerance dose. Some experimental infection results suggest that the distribution of the tolerance dose can be described log-normally [62, 63, 66]

(5) $$\begin{array}{c} P\left(Z\right)=\frac{1}{\sqrt{2\pi }}\overset{Z}{\underset{-\infty }{\int }}\exp\left(\frac{x^{2}}{2}\right)dx, \end{array}$$

(6) $$\begin{array}{c} Z=\frac{\ln N-\mu }{\sigma }, \end{array}$$

where *P*(*Z*) is the frequency distribution of the tolerance dose, *Z* is the normalized tolerance dose, *N* is the intake dose, and *μ* and *σ* are the mean and standard deviation of natural logarithm of the tolerance dose, respectively. These statistics are determined by fitting the infection dose data for a pathogen in an experiment. Sze-To and Chao [62] consider Weibull distribution as another possibility.

Stochastic interpretations of the dose-response model also exist. Due to mathematical complexities, these models predict infection risk only for one suspect (as opposed to a population). Generally, the greater the intake dose is, the greater the probability of infection will be. In stochastic single hit models, the host must intake a dose containing at least one pathogen that reaches the infection site and survives until symptoms develop. For aerosolized pathogens, exponential and Beta-Poisson models have been suggested [62, 63]. The exponential model is given by

(7) $$\begin{array}{c} P_{I}=1-\exp\left(-rN\right), \end{array}$$

where *r* is infectivity of pathogens and *N* is the intake dose. If there is only one available infection dose value, only the exponential model can be used, as the other models require at least two infectious dose values to calculate the fitting parameters.

Sze-To and Chao [62] have developed an infection risk model that can incorporate aerosol size and spatial and temporal factors into a dose-response model. By this approach, a model can be formed that gives the airborne infection risk for a subject (moving or stationary) as a function of time. The exposure level of the pathogen at location *x* and during time interval *t*_0_ is given by

(8) $$\begin{array}{c} E\left(x,t_{0}\right)=cp\overset{t_{0}}{\underset{0}{\int }}v\left(x,t\right)f\left(t\right)dt, \end{array}$$

where *c* is the pathogen concentration in the respiratory fluid (i.e., oral mucus and saliva), *f*(*t*) is the viability function of the virus or bacteria in the aerosols, and *v*(*x*, *t*) is the volume density of expiratory droplets at the location. *v*(*x*, *t*) can be obtained by CFD modeling. As it is a time consuming computation to determine exposure levels for every expiratory action (like a cough or a sneeze) and in all locations, one can compute the exposure level for one expiratory action and then multiply the exposure level by the number of expirations during the exposure time interval. By this approach, a stochastic and nonthreshold dose-response model for the airborne infection risk can be formed

(9) $$\begin{array}{c} P_{I}\left(x,t_{0}\right)=1-\exp\left(-\overset{m}{\underset{j=1}{\sum }}r_{j}\beta_{j}f_{s}cp\overset{t_{0}}{\underset{0}{\int }}v{\left(x,t\right)}_{j}f\left(t\right)dt\right), \end{array}$$

where *m* is the total number of aerosol size bins, *v*(*x*, *t*)_*j*_ is the volume density of droplets of the *j*th size bin, and *f*_*s*_ is the expiration frequency. As the infectivity (reflected in *r*) and deposition fraction of infectious aerosols (reflected in *β*) are aerosol size dependent, *v*(*x*, *t*) is thus split into different size bins.

#### 2.5.3. Population Infection Model

Some studies in the literature model airborne infection risk for a *population* of individuals, as opposed to a single suspect [65, 67, 68]. Such models are termed *population* or *epidemic* infection models and simulate dynamics in a total population (*N*) that consists of the susceptible (*S*), infected (*I*), and recovered (*R*) subpopulations [3, 65]. The relationships among these subpopulations are expressed using a series of differential equations that relate physical and biological parameters. The model complexities depend on the number of parameters considered. Various researchers have considered aerosol size, probability of infection by an inhaled pathogen, physical removal of airborne pathogens, infection recovery rate, inactivation rate of airborne pathogens, airborne pathogen generation rate, and many more parameters [3, 4, 65, 67, 68]. For example, the work of Noakes et al. [3] has integrated the Wells-Riley model into a *population* infection model. Noakes and Sleigh [4] have also developed a *population* model that finds infection rate for a multizone health care facility.

Although these models extend airborne infection risk prediction to a population, they do not resolve infection risk spatially, since they assume well-mixed distribution for airborne pathogens. Inclusion of spatial resolution will result in many mathematical complexities for such models. On the other hand, the Wells-Riley (1) and dose-response models (e.g., (5), (6), and (7)) predict the infection probability for a single suspect, but they can resolve spatial and temporal components of risk (e.g., (8) and (9)) and hence suffice to guide ventilation design.

## 3. Impact of Building Ventilation Design Parameters on Airborne Infection Risk

### 3.1. Categories of Ventilation Systems

Ventilation systems can be classified according to the mechanisms driving airflow. *Mechanical ventilation* systems are fan driven. *Positive pressure* mechanical ventilation systems are fanned on intake and result in exfiltration of space (i.e., air tends to leak out of ventilated space). On the other hand, *negative pressure* mechanical ventilation systems are fanned on exhaust and result in infiltration of space [5, 69].

*Natural ventilation* systems rely on natural forces such as wind or a density-generated pressure differences between indoor and outdoor to drive air through building openings. Some purpose-built openings include doors, windows, solar chimneys, and wind towers [5, 69]. Advanced natural ventilation systems with passive cooling or heating have also been developed. In these systems, outdoor air is supplied via stacks fed from below-ground concrete plena providing passive cooling or heating. Air leaves the space through stacks. In some advanced systems, central control units operate dampers at inlet and outlet locations for each space [69].

*Hybrid (mixed-mode) ventilation* systems rely on natural driving forces when sufficient and use mechanical ventilation for augmentation as necessary. Usually, exhaust fans are used to assist natural ventilation in a negative pressure arrangement [5]. In some systems, passive down-draught cooling (PDC) encourages air to fall through chilled water pipes at a high level during hot weather. During cool weather some systems use exhaust flows to warm the incoming air. Usually, sensors and control technologies are required for optimum performance of hybrid systems [69].  Table 7 shows major advantages and disadvantages associated with the ventilation systems described above [5].

Another categorization for ventilation type relates to the structure of the air motion. Two important variations are mixing and displacement ventilation systems. *Mixing* ventilation aims at creating a uniform low concentration of infected air that is subsequently extracted. The air is supplied along the ceiling with high turbulence for effective mixing [10]. *Displacement* ventilation flows are driven by air density differences in the room (buoyancy). In practice, neither pure mixing nor pure displacement ventilation can be achieved. There is always a combination of the two mechanisms with one being dominant [10]. 

### 3.2. Ventilation System Performance

#### 3.2.1. Air Exchange Rate

Air changes per hour (ACH) is defined differently for positive and negative pressure rooms. For positive pressure rooms, it is the ratio of the volume of outdoor air flowing into a given space in an hour divided by the volume of that space. For negative pressure rooms the exhaust airflow rate is used for this calculation [5]. Typically, a higher ACH results in more dilution of pathogens and reduced airborne infection risk [5, 69]. If the outdoor conditions are favourable (e.g., temperature differences and wind patterns), naturally ventilated buildings have higher ACH than mechanically ventilated buildings.

Most building codes mandate a minimum ACH to prevent airborne disease transmission by sufficient air dilution. For example, US center for disease control (CDC) and prevention, world health organization (WHO), and the american society of heating, refrigeration, and air-conditioning engineers (ASHRAE) all require a minimum of 12 ACH and negative pressure for newly built airborne infection isolation rooms [70–72].  Table 8 shows a list of health care facility functional spaces, with examples of subspace ACH and pressure requirements [72].

Standard 170 of the American Society of Heating, Refrigerating, and Air-conditioning Engineers (ASHRAE) defines various functional spaces in health care facilities and specifies the minimum ACH in each space (see Table 8) [72].

*Critical care* units such as wound intensive care units (e.g., burn units) are required to be provided with individual humidity control. *Airborne infection isolation* (AII) rooms are defined as spaces to isolate patients with highly infectious diseases (e.g., *Tuberculosis* and influenza). For these rooms, the code requires continuous negative differential air pressure with the exhaust directed to the outside without mixing with other exhaust streams in the facility. Further, the exhaust position is required to be above the patient's bed. *Protective environment* (PE) rooms are designed to protect immunocompromised patients (e.g., AIDS) from human and environmental airborne pathogens. The code requires these rooms to be well sealed and to provide continuous positive differential air pressure. Also, the air inlet diffuser is required to be above the patient's bed, and the exhaust return is required to be near the door. *Surgery* rooms are classified in three major subcategories (A, B, and C). Class A surgery provides minor surgical procedures without preoperative sedation. Class B surgery is minor surgery with oral, parenteral, or intravenous sedation or under analgesic or dissociative drugs. Class C surgery provides major surgical procedures that require general or regional block anesthesia and support of vital bodily functions. The code requires positive pressure differential for class B and C surgery rooms. In addition, the inlet diffusers should be placed on top of the surgical bed, and the return exhaust grilles should be near floor level. For *Morgue* and *Autopsy* rooms, the code requires that the exhaust air should not be combined with air from any other exhaust systems.

Contrary to the ASHRAE 170 standard, which requires mechanical ventilation in all functional spaces, other standards have promoted the use of natural ventilation. For example, the United Kingdom national health service (NHS) policy mandates mechanical ventilation only for principal medical treatment areas such as airborne infection isolation rooms, operating theatres, and associated rooms. Inpatient rooms are not required to be ventilated mechanically [5].

#### 3.2.2. Temperature and Relative Humidity

As discussed in Section 2.3.2, temperature and relative humidity affect pathogen viability. The building code has not yet been fully refined to require specific combinations of temperature and relative humidity to reduce airborne infection risk. Neither does it make any recommendations specific to the type of pathogen that is being considered. For example, ASHRAE 170 requires a temperature range of 20°C –26°C and a relative humidity range of 30%–60% for most health care functional spaces [72]. Part of the difficulty is the lack of knowledge for aerosolized pathogen survival behavior in various environmental conditions. Also, some environmental conditions are against human comfort or the recovery process. More research is needed in this field to improve the building code.

Another area of concern is microbial growth in humid environments within the HVAC system (e.g., ducts, humidifiers, evaporative air coolers, cooling coil drain pans, and condensation sites). Kowalski and Bahnfleth [12] report that spores in particular take advantage of humid conditions to germinate and multiply. ASHRAE 170 limits the amount of relative humidity to a maximum value of 90% throughout the duct work of any HVAC system [72].

#### 3.2.3. Airflow Distribution Structure

Increasing ACH (higher dilution) is not the only way to reduce airborne infection risk. ACH is a useful but blunt instrument to assess the ventilation rate of a space. A room may have a high overall ACH but with low ventilation in specific areas. Various formulations have been developed to account for this difference. For example, Chung and Hsu [73] define *ventilation effectiveness* that gives a spatial resolution to local air refresh rates

(10) $$\begin{array}{c} E_{i}=\frac{C_{e}-C_{s}}{C_{i}-C_{s}}, \end{array}$$

where *E*_*i*_ is the relative ventilation effectiveness at location *i*, *C*_*e*_ is contaminant concentration in the exhaust, *C*_*s*_ is the contaminant concentration in the supply diffuser, and *C*_*i*_ is the contaminant concentration at location *i*. A higher *E*_*i*_ indicates a higher ventilation effectiveness. Rooms with short-circuited airflow patterns will have very high ventilation effectiveness in some areas, while stagnant air in other areas corresponds with very low ventilation effectiveness [5]. A complete ventilation study must consider both ACH and space-resolved air exchange effectiveness.

The local air exchange effectiveness requirements have not yet been standardized in most building codes partly because the interdependencies of air exchange efficiency with other design parameters are not well understood. For example, one would need to study the room interior decoration, placement of exhaust and diffuser, occupancy, and infection spread simultaneously to make the necessary recommendations. Recently, as it will be discussed in Section 3.3, much research has been focused on studying the impact of airflow distribution structure on dispersion of airborne disease agents and subsequently infection risk.

The type and location of air supply and removal systems are key factors affecting the airflow distribution structure in ventilation spaces. For mechanical ventilation, ASHRAE defines various air supply diffuser types according to Table 9.

In mechanical ventilation, one strategy to reduce infection risk is to use displacement ventilation. The vertical upward type displacement ventilation introduces fresh cool air near the bottom. The air temperature rises by the heat from warm objects (like human bodies), and the buoyant force takes the warm and polluted air (possibly containing airborne pathogens) close to the ceiling and subsequently the exhaust for removal [10]. Downward displacement ventilation, on the other hand, introduces cool and heavy air at the top with the exhaust removal at the bottom. The cool air drops due to negative buoyancy and reaches the floor if air mixing is avoided. This ventilation scheme, if it could be properly designed and operated, would be ideal for removing large droplets [10, 36]. For upward displacement ventilation, high ceilings are required and the heat gains by walls and equipments must be minimized. For downward displacement ventilation, air mixing should be avoided as much as possible.

ASHRAE 170 [72] strictly bans the use of upward displacement ventilation in all health care facilities. For example, it requires only group E diffusers for all class surgery rooms, Protective environment (PE) rooms, wound intensive care units, and group A or E diffusers for airborne infection isolation rooms. The principal guideline by ASHRAE is to increase airborne pathogen dilution and hence to reduce infection risk. Other guidelines allow for displacement ventilation in health care facilities. For example, CDC [70] suggests downward displacement ventilation for isolation wards.

Also, the location, size, and volumetric airflow of supply and extraction openings affect flow patterns and airborne infection risk levels. The arrangement of inlet and outlet openings can cause different flow recirculation scales which may change the mean age of indoor contaminants. A useful technique for optimizing the properties of inlet and outlet openings is to use tracer gas experiments and calculate ventilation effectiveness at different sites.

For naturally ventilated buildings, the prediction of airflow distribution structure is more difficult, since outdoor air movement behavior is less predictable. Two major factors affecting this are wind pressure and stack (or buoyancy) pressure. When wind strikes a building, it induces a positive pressure on the windward face and a negative pressure on the leeward face. This drives airflow through the building from the positive to the negative pressure openings. The wind pressure is standardized by the dynamic pressure of the outdoor wind speed [5]

(11) $$\begin{array}{c} C_{P}=\frac{P_{T}-P_{\text{AS}}}{\left(1/2\right)\rho V_{H}^{2}}, \end{array}$$

where *C*_*P*_ is wind pressure coefficient, *P*_*T*_ is total pressure, *P*_AS_ is atmospheric static pressure at the building height, *ρ* is density of air, and *V*_*H*_ is wind velocity at far field.

The stack (or buoyancy) pressure is generated by the air temperature (or density) difference between indoor and outdoor air. This difference generates an imbalance in the pressure gradients of the inside and outside air columns causing a vertical pressure difference. The ventilation rate through a stack is a function of the pressure differential between two openings of the stack [5]

(12) $$\begin{array}{c} \Delta P_{S}=\rho_{o}gH\frac{T_{i}-T_{o}}{T_{o}}, \end{array}$$

where *P*_*S*_ is stack pressure, *ρ*_*o*_ is density of outdoor air, *g* is gravitational acceleration, *H* is height between two openings, *T*_*i*_ is indoor temperature, and *T*_*o*_ is outdoor temperature.

Natural ventilation systems can be categorized into four groups. In *cross flow* systems, there are no obstacles on either side of the prevailing wind. In *wind tower* systems, the wind is caught on the positive pressure side and extracted on the negative pressure side. In *simple flue stack* systems, a vertical stack at each room allows for air movement to the roof. In a *solar atrium stack*, a large stack is heated by solar radiation, assisting the air movement and removal in the upward direction [5].

Hybrid ventilation system design methods can be grouped into three major categories. In *fan-assisted stack* systems, a fan supplements the extraction of air at the exhaust location of stack. In *top-down* systems, the fan extraction is assisted by a wind tower. In *buried pipe* systems, when land is available, ventilation pipes (ducts) are used to bring air temperature to steady-state values [5].

Major design elements for natural or hybrid ventilation systems require *site analysis, building design analysis, and vent opening design*. The site analysis concerns building location, layout, orientation, and landscaping; building design analysis involves the type of building, functions, form, envelope, internal distribution of spaces, and thermal mass; vent opening design concerns the position, type, size, and control of openings [5].

Occupation density is an important factor affecting airflow distribution structure in a space. Overcrowding is often correlated with increased rates of infection. Not only the high thermal load of people will make the airflow distribution structure unpredictable, but also droplet mode and contaminated surfaces will contribute towards disease transmission. Some physicians believe there should be a minimum of two arm lengths between patients [7].

Human respiratory activities also affect the airflow distribution structure. Breathing, coughing, and sneezing may all affect the room airflow. Using CFD studies, Gao and Niu [35] demonstrate that normal breathing does not dramatically perturb the airflow in ventilated spaces. However, the interaction between coughs and sneezes with the background airflow can be complex and needs to be studied in detail.

Human occupants also generate thermal plumes that interact with ventilation airflow and other plumes in ventilated space. In some situations (e.g., upward displacement ventilation), these plumes can assist aerosol removal. Thermal plumes can also prevent aerosol removal from ventilated spaces. For example, a malfunctioning upward displacement ventilation system can create recirculation zones that keep aerosols in the domain for long periods of time. Also, thermal plumes increase mixing in downward displacement ventilation systems, impeding aerosol removal through low level exhausts [36].

Occupants' motion and other activities also perturb the airflow in the ventilation domain. For example, the wake of a person when walking from one location to another may be responsible for mixing of air and hence increasing airborne infection risk. Other actions such as opening or closing doors and windows can also be important [10]. In general, the impact of occupancy is poorly understood, and much research is needed for the improvement of building codes.

### 3.3. Experimental Studies of Aerosol Dispersion in Different Ventilation Systems

Wan et al. [34] performed CFD simulations and IMI experiments to determine expiratory droplet dispersion in a hospital ward with mixing type ventilation system. They used an air-blast nozzle for droplet generation and injection. This nozzle provided an injection velocity of 10 m/s, an air temperature of 307 K, and an airflow rate of 0.4 L/s. The volatile fraction of the surrogate fluid was 0.94 that was representative of oral fluid. In their simulations, they used droplets of various size bins in the range of 1.5–1500 *μ*m with a mode of 12 *μ*m. For a perfectly mixed system, the decay of aerosols due to removal in a ventilation system is exponential. They found, however, that decay rate is faster in reality due to deposition of droplets and the fact that most mechanical ventilation systems operate in conditions between perfectly mixed and perfectly displaced ventilation. Two distinctive behaviors were observed: small size group aerosols (<45 *μ*m) exhibited airborne transmittable behavior, while large size group aerosols (>87.5 *μ*m) settled quickly with gravitational force. Also, the dispersion of droplets exhibited different regimes with elapsed time. This was due to momentum interaction of the jet with the background flow and also the size change of aerosols due to evaporation.

Zhang and Chen [30] studied the dispersion of fine monodisperse and nonevaporating aerosols in ventilation systems with ceiling supply, side wall supply, and underfloor airflow distribution systems. A condensation aerosol generator was used to generate aerosols in the range of 0.31–4.5 *μ*m. A particle counter was used to measure concentration at different heights. They observed that the underfloor system has a better aerosol removal performance than the ceiling type and side wall supply systems.

Yin et al. [74] studied dispersion of tracer gas and fine monodisperse nonevaporating aerosols in mixing and displacement ventilation systems for mock-ups of fully occupied hospital wards. They used a tracer gas (*SF*_6_), 1 *μ*m and 3 *μ*m aerosols that were released at steady rate at patient bed. A photoacoustic multigas analyzer with a multipoint sample was used to measure *SF*_6_ concentration at various locations. An APS was used to measure the aerosol concentration. It was shown that the displacement ventilation with 4 ACH removes tracer gas and fine aerosols more effectively than the mixing type ventilation with 6 ACH. They observed that the performance of the displacement ventilation system is very sensitive to the location of all exhausts. If any exhaust is located at low level, the pollutant concentration at breathing zone will be worse than mixing type ventilation. All exhausts must be located at high levels, preferably closer to the pollutant source.

Chung and Hsu [73] studied the effect of diffuser and exhaust locations on the removal of contaminants. They used CO_2_ tracer gas in the supply and observed how ventilation effectiveness (10) changes at various locations. Interestingly, although the total air exchange rate is insensitive to the location of the diffuser and exhaust, the local ventilation effectiveness varies greatly from one case to another. For example, excluding the effect of occupancy and buoyancy, the most efficient design is to locate diffuser and exhaust face to face at the same height and at opposite sides of the room. The complexities arise for actual rooms with occupancy and heat gains, in which scenario a detailed study must be performed.

A study by Qian et al. [36] used N_2_O tracer gas to observe the effect of diffuser and exhaust locations on the performance of ventilation system to remove pollutants in a mock-up hospital ward with thermal manikins. In their experiments, the diffuser was placed over the patient's head, but the location of the exhaust was varied. All experiments were run at 4 ACH. They observed that downward ventilation system could not produce a unidirectional airflow pattern, since thermal plumes of manikins induced mixing and disturbed pollutant removal. On the other hand, a higher location for the exhaust caused more effective removal of pollutants.

A study by Escombe et al. [64] in Peruvian hospitals in Lima revealed that opening doors and windows could provide a median ventilation of 28 ACH for inpatient rooms. They also report that facilities built more than 50 years ago, with large doors, windows and high ceilings, provided a median ventilation of 40 ACH. They used CO_2_ tracer gas experiments to demonstrate high air exchange rates for natural ventilation in ideal conditions.

A study by Yu et al. [75] shows that naturally ventilated high-rise buildings with interconnected flats benefit from high ACHs, but the airborne pathogens can travel between flats, usually to higher levels where the infection risk will be highest.

### 3.4. Engineered Disinfection of Air

#### 3.4.1. Filtration

In general, two sources of clean air can be used to refresh indoor air: outside air and filtered air. Sometimes a combination of these are also used. Conditioning outside air can be energy intensive, but, on the other hand, using filters in HVAC systems provides substantial opportunities to save energy in buildings. Kowalski and Bahnfleth [12] show that under certain conditions, using recirculated air with high-efficiency particulate air (HEPA) filters reduces particulate concentration for indoor air similar to full outside air systems. Cole and Cook [11] also report that ventilation plus recirculating air filtration could reduce droplet nuclei concentrations with 30%–90% effectiveness.

Minimum efficiency reporting value (MERV) rating is a measurement scale designed by ASHRAE to rate the effectiveness of air filters.  Table 10 shows different classes of MERV rating, each designed to filter aerosols in a specific range.

ASHRAE 170 requires up to two filter banks in the design of any health care ventilation system. Filter bank no. 1 is placed upstream of the heating and cooling coils and the supply fans to filter all of the incoming mixed air. Filter bank no. 2 is installed downstream of all wet air cooling coils and supply fans.  Table 11 shows the required MERV rating and filter banks for some critical functional spaces in health care facilities [72].

One difficulty with higher MERV rating filters (e.g., HEPA) is blockage and the necessity to clean or replace them on a regular basis [11]. Sometimes medium MERV rating filters are used instead while they are still capable of removing airborne pathogens, especially spores, without high operation costs. The overall removal efficiency of the filtration system is improved if the filter is placed in the recirculation loop instead of the outside air intake or downstream of the cooling coils [12].

A few challenges remain in the filter making technology. For example, more efficient filters cause more pressure drop, and hence require auxiliary fans or air pumps to supply the required pressure. This increases energy consumption of the HVAC system. Also, high-efficiency filters are difficult to use with natural ventilation systems, since the pressure differential in such systems is not enough to drive flow through such filters. The other challenge is filtration of aerosols in the range of 0.1–0.3 *μ*m with economic solutions. Aerosols smaller than 0.1 *μ*m are efficiently captured by diffusional forces, and aerosols larger than 0.3 *μ*m are efficiently captured by impaction.

#### 3.4.2. UV Radiation

Use of ultra violet (UV) radiation can play a key role in disinfecting pathogens or limiting their growth. For example, UV radiation has been used to limit microbial growth in cooling coils. UV radiation impairs fungal growth and in some cases kills spores. Key factors to consider are air velocity, local airflow patterns, degree of maintenance, resistance of microbes, and humidity. Chronic dosing with UV radiation can also have a major impact on disinfecting airborne viruses and bacteria. One-pass exposure of pathogens to UV light may not be effective to disinfect them, but recirculating air through the UV radiation unit can be very effective to disinfect the air [11, 12]. UV disinfection equipment is either placed upstream of air supply system or within the ventilated space close to the ceiling, where human exposure is minimal.

### 3.5. Architectural Programming

#### 3.5.1. Source Management

Infectious pathogens usually come from a source, either a human being or an object. These sources can be managed by segregation of human sources and also regular cleaning and inspection of materials. For example, patients with *Tuberculosis* can be housed in negative pressure rooms, required to wear masks, or placed in laminar flow beds until recovered. Further, the room layout (e.g., bed placement, door and window operation, and interior decoration) and division of floor plan into subspaces connected by corridors can be designed carefully to segregate pathogen sources from suspects. As far as regular cleaning and inspection are concerned, mold-contaminated building materials can be purged by hot water systems, and filters and HVAC units can be regularly inspected and cleaned to eliminate surface sources. Recent research also shows a positive correlation between airborne or surface dust mass and corresponding fungi, bacteria, and viruses, hence even dust control can be a preventive measure in reducing pathogen sources [11].

#### 3.5.2. Activity Management

Activity management is the process of ensuring that a building is used in a way it was designed for. Sometimes, airborne infection risk increases because the building functional spaces are overpopulated. Other times, it is the result of a section of the building being used for other reasons than for which it was designed in the first place. Examples include laboratories in structures that were designed to be offices or living quarters or offices that were originally designed as facilities. Another possibility is the alteration of the built environment by shuffling furniture or adding extensions to an already designed space [11].

## 4. Conclusions and Research Needs

Past outbreaks of disease transmission in health care facilities have identified many pathogens such as *Tuberculosis*, influenza, and *Aspergillosis*, whose spread is strongly linked to airborne transmission. As a result, there is renewed interest in prevention of airborne disease transmission in health care facilities. Research has led to the development of useful methods for the prediction of various aspects of airborne disease transmission. Physical modeling of, and experiments on, aerosol transport have revealed mechanisms for dispersion, heat, and mass transfer of aerosols in different size regimes from submicrometer to millimeter. Many detailed infection models such as Wells-Riley model (1) and dose-response model (e.g., (5) to (9)) have been suggested for prediction of the spread of airborne disease in health care facilities. The current state of research has also shown that important sources for airborne pathogens including human activity (expiratory, resuspension, and shedding) and building microbial ecology (indoor environment and HVAC components). The viability and infectivity response of various airborne pathogens to environmental conditions, such as temperature and relative humidity, have also been understood and quantified in considerable depth. For example, animal tests, culture methods, molecular methods, and plaque assay methods have been used to measure pathogen viability and infectivity decay rates for many different classes of microbes although all these techniques suffer from substantial shortcomings. The effect of different ventilation strategies (mixing, displacement, and natural) on air exchange rates and the spread of aerosols has also been studied. For example, mixing type ventilation can achieve sufficient dilution of contaminated air to reduce the infection rate. A displacement ventilation strategy, if implemented successfully, helps segregate contaminated from noncontaminated air, thus reducing the infection risk. Under ideal conditions, natural ventilation has been shown to exhibit very high air exchange rates (and thus dilution) that reduce the airborne infection risk. In addition, various building codes have implemented preventive measures to reduce the airborne infection risk. For example, high efficiency filtration and high air exchange rates are mandated for critical care units (surgery and protective environment rooms), and exhaust outlets are recommended to be installed close to contaminant sources.

Current methods can aid ventilation design for healthier buildings. However, despite recent advances, there is much to be researched to improve these methods. The improvements can be realized considering the same infection pathway presented in Figure 1 and influences that the environmental and engineering controls might have on each step along the pathway. The sections below suggest future research needs in ventilation design for health care facilities in the order of steps in the infection pathway.

### 4.1. Relative Importance of Airborne versus Other Infection Pathways

Efficient ventilation design for health care facilities is possible only if the relative importance of the airborne infection pathway is known for numerous pathogens. In other words, one needs to know the importance of all pathways (direct and indirect) to justify a particular ventilation design in the first place. The direct pathway for infection (e.g., shaking hands, close contact, etc.) does not require a fluid carrier phase (air) to transport pathogens from the source to the receptor over several minutes, and hence, ventilation design has no effect in its mitigation. If the direct contact mode for infection is primary for a certain disease, increasing the air exchange rate will achieve nothing but added energy cost to a building. The airborne infection pathway is considered to be important for some pathogens such as *Tuberculosis* and influenza. However, there is still uncertainty about the relative importance of different infection pathways for many other diseases. The reason for the uncertainty is the lack of data analysis in actual outbreaks and ethical concerns about running relevant experiments. Documentation, analysis, and interpretation of historical data in infection spread is a useful tool to reveal the importance of airborne mode of infection for many diseases.

### 4.2. Source Management

The proper design of procedures in a health care facility is perhaps the most effective approach in preventing airborne infection risk. Future research should recommend what medical procedures minimize exposure to airborne pathogens. This may include physical separation of the infected population from others. Even if this is not completely possible (true for caregiving staff), recommendations regarding the use of personal protective gear, hygiene procedures (e.g., washing hands), and mechanics of providing care can be very helpful. In addition, hospital admissions and care provision methods can be specific to the type of infection considered. The building code requirements can improve significantly to include disease-specific recommendations.

### 4.3. Source Characterization

Human activities are important sources for generation of pathogenic aerosols. Respiratory expirations (speech, cough, and sneeze) generate many viable and infective pathogens from submicrometer to millimeter size. Also, skin shedding, walking, and other nonexpiratory activities generate or resuspend pathogenic aerosols. Although it is known that expiratory activities disperse pathogens such as influenza and *Tuberculosis*, it is still an open question whether the generation of other pathogens by expiration is also important.

### 4.4. Dispersion

#### 4.4.1. Effect of Ventilation Design on Airflow Distribution Structure

Most current building codes do not explicitly take into account the airflow distribution structure in mandating ventilation design. Most efforts to reduce airborne infection risk are limited to increasing air exchange rates and higher dilution of contaminated air. Even when specific recommendations are made regarding placement of inlet diffusers and outlet exhaust locations, the guidelines are too general. Each ventilation strategy (i.e., mechanical, displacement, or hybrid) results in a certain airflow distribution and ventilation effectiveness (10) for the whole spatial domain. There is much research to be performed to reveal the effect of room envelope conditions, the location of diffusers and exhaust ports, the air exchange rate, and the ventilation strategy used. It is true that such analysis is very specific to the type of room being designed, and detailed CFD simulations may be necessary, but the building code can allow for such analysis in the design of ventilation systems for a room. Specific requirements can be made to demonstrate ventilation effectiveness in areas of concern in a room (near contaminant source and away from it, where suspects may be exposed to pathogens).

#### 4.4.2. Effect of Occupancy on Aerosol Transport

The effect of occupancy on ventilation of health care functional spaces has not been researched in detail. Occupancy affects the airflow distribution structure, and therefore, the airborne infection risk significantly. The occupational density of people, and also their location in a room relative to the room's interior decoration, diffuser, and exhaust can either reduce or increase airborne infection risk. People introduce thermal plumes in the ventilation space, and, in general, if thermal plumes in a room increase flow circulation and mixing, it will increase the airborne infection risk. In addition, the motion of people, and associated functions such as opening doors or windows, alters the ventilation flow pattern in a room. Such effects must carefully be considered and modeled to arrive at occupation-specific recommendations in building code.

#### 4.4.3. Interaction between Expiratory Aerosols and Ventilation Airflow

The interaction of expiratory pathogens with ventilation airflow needs to be researched in greater detail. The momentum (breathing versus sneezing) and direction (vertical versus horizontal) of human expirations disturb the ventilation airflow and need to be considered for effective ventilation design.

#### 4.4.4. Prediction of Airflow Structure

The room airflow structure can have a major impact on infectious aerosol concentration (factor of two or more) beyond the simple effect of increased ventilation rate. However, to improve the airflow structure, it is required to use CFD simulations. There are many improvements to be made in the simulation methods.

One limiting factor in predicting the airflow structure in ventilated space is related to air turbulence. Current economic turbulence models (RANS and DES) predict some qualitative aspects of airflow in the ventilated space, but more accurate modeling of turbulence is possible only using more computationally intensive models (LES and DNS). Aerosol transport is particularly affected by larger eddies, a feature of the ventilation flow that RANS models cannot predict accurately. With increased computational power and more advanced CFD simulations, the inclusion of more accurate turbulence models in ventilation airflow simulations can predict the chaotic behavior of airflow in greater detail and, hence, predict airborne infection risk more accurately.

Although there are improvements to be made in the simulation methods, the models are extremely sensitive to initial and boundary conditions. Some of this uncertainty is irreducible. For example, people cough or sneeze with unpredictable directions, strengths, and locations. Further, day-to-day variations in room use and weather conditions introduce additional variations in aerosol transport. This complexity implies the need to simulate a very large number of cases to assess room performance, but fortunately, computing power is becoming inexpensive enough to contemplate integrating such simulations into the building design process.

### 4.5. Viability and Infectivity

#### 4.5.1. Pathogen Response to Environmental Conditions

The effect of environmental factors, such as temperature and relative humidity, on the survival of some aerosolized pathogens has been studied. However, the literature is limited to a few diseases, and a large class of aerosolized pathogens are yet to be analyzed. The effect of OAF (relying on natural ventilation) and electromagnetic radiation (relying on daylighting and UV disinfection) on the survival of pathogens need to be studied in greater detail. As far as the building code is concerned, the recommendations for temperature and relative humidity in functional spaces of health care facilities are very conservative. Future research should reveal more detailed mechanisms for pathogen survival behavior as a function of temperature and relative humidity so that the building code will recommend more specific environmental conditions to reduce airborne infection risk in a pathogen-specific fashion.

#### 4.5.2. Development of Real-Time Viability Detecting Devices

Animal tests, culture methods, molecular methods, and plaque assay methods have come a long way to provide practical experimental protocols for viability and infectivity measurements of aerosolized pathogens. However, each of these methods is limited with its own advantages and disadvantages so that no particular protocol provides all the desired information. All of these protocols share a common disadvantage which is that they take a long experimental time (hours if not days) to arrive at desired results. Even if compromises are to be realized, the research is yet to provide standardized tests that allow real-time detection of aerosolized pathogen viability and infectivity measurements.

### 4.6. Air Disinfection Technologies

New trends in building ventilation design emphasize engineered air disinfection techniques including filtration and UV radiation. These techniques are usually more cost effective compared to increasing building ventilation rates. The effect of these technologies on pathogen survival behavior should be studied in greater detail so that effective ventilation design of health care facilities will be possible. For example, UV radiation targets genomes in pathogens, while pathogens adapt to the environment by genetic mutation, a phenomenon that is less understood. Pathogen interaction with filters should be studied in greater detail to observe whether filtration actually disinfects air or provides a growth medium for it. Also, filtration technology needs to improve for removing aerosols in the range of 0.1–0.3 *μ*m efficiently.

### 4.7. Infection Mechanism

A particular challenge in predicting airborne infection risk is the lack of knowledge for actual infection mechanisms and infection dosage. The airborne infection involves deposition of aerosolized pathogens on infection sites in a host and the ability to multiply and target specific cells until infection symptoms develop. Another difficulty is the lack of experimental data for infection. Most infection outbreaks in history provide limited information, making it difficult to fully analyze and arrive at the desired conclusions. Even empirical quanta estimations in well-mixed rooms using the Wells-Riley model (1) combine various complicated and interconnected factors. Such empirical data cannot be used in more detailed models of infection risk, where spatial distribution of risk, subject immunity variation, and deposition mechanisms are to be resolved. As a result, many such models become probabilistic with simplifying assumptions (e.g., single hit model) to quantify risk. Any experimental infection test is valid only for the particular setup of the experiment and is difficult to generalize to provide a universal infection dosage or mechanism. Future research should set a standard procedure to establish a minimum infection dosage. Even if there are compromises to be made (e.g., minimum dose for animal infections), there are useful merits to such a standardization.

### 4.8. Spatial and Temporal Resolution of Infection Risk

The most widely used engineering tool to aid building code design at the moment is the Wells-Riley infection risk model (1) for the well-mixed room. Although useful, this model recommends nothing but increasing air exchange rate to reduce airborne infection risk. Engineering research has come a long way to model airborne infection risk with spatial and temporal resolution. Current successful models are stochastic in nature (e.g., (5) to (9)) but can still provide useful guidelines for ventilation design, especially when airflow distribution structure is to be accounted for. Future research in the development of these models for various pathogens can improve the ventilation design of health care facilities.

## Figures and Tables

**Table 1:** Experimental expiratory droplet size data.

Study	Measurement technique	Expiration type	*D*_min⁡_ [*μ*m]	*D*_max⁡_ [*μ*m]	Geometric mean [*μ*m]	Geometric standard deviation [*μ*m]
Duguid [14]	Microscopy	Coughing	1	2000	14	2.6
Duguid [14]	Microscopy	Sneezing	1	2000	8.1	2.3
Loudon and Roberts [19]	Microscopy	Coughing	1	>1471	12	8.4
Papineni and Rosenthal [16]	OPC^1^	Talking	<0.6	2.5	0.8	1.5
Papineni and Rosenthal [16]	OPC	Nose breathing	<0.6	2.5	0.8	1.5
Papineni and Rosenthal [16]	OPC	Mouth breathing	<0.6	2.5	0.7	1.4
Papineni and Rosenthal [16]	OPC	Coughing	<0.6	2.5	0.7	1.5
Papineni and Rosenthal [16]	ATEM^2^	Mouth breathing	<0.6	2.5	1.2	1.6
Chao et al. [18]	IMI^3^	Talking	2	2000	12.6	3.2
Chao et al. [18]	IMI	Coughing	2	2000	13.1	3.6

^1^OPC: optical particle counter, ^2^ATEM: analytical transmission electron microscope, ^3^IMI: interferometric Mie imaging.

**Table 2:** Summary of turbulence modeling approaches (with representative number of required computational cells and computational time to simulate one hour of ventilation flow in a hospital inpatient room).

Turbulence model	Advantages	Disadvantages	Cells	Time
DNS^1^	Resolves eddies of all lengths	Computationally very expensive	10^10^	Years
LES^2^	Resolves large eddies	Computationally expensive	10^8^	Months
DES^3^	Computationally economic	Difficult to implement	10^7^	Weeks
RANS^4^	Computationally economic	Less accurate, difficult to converge	10^6^	Days

^1^DNS: direct numerical simulation, ^2^LES: large eddy simulation, ^3^DES: detached eddy simulation, ^4^RANS: Reynolds averaged Navier-Stokes.

**Table 3:** Summary of viability and infectivity measurement techniques for airborne pathogens.

Measurement technique	Advantages	Disadvantages
Animal tests	Common diseases between humans and animals and interaction with the host	Difficult to extrapolate test results to human infection
Culture methods	Reproducibility	Pathogen interaction with host
Molecular methods	Single-pathogen detection limit	Reproducibility and pathogen interaction with host
Plaque assay methods	Infectivity and interaction with live cells	Interaction with host

**Table 4:** Summary of most probable target molecules [43].

Stress	Most probable target molecules
Relative humidity and temperature	Outer membrane lipids and proteins
Oxygen	Lipids and proteins
Ozone	Lipids and proteins
Open air factor (O_3_ + olefins)	Lipids, proteins and nucleic acids
*γ*-rays, X-rays, and UV radiation	Lipids, proteins and nucleic acids

**Table 5:** Summary of the effect of temperature and relative humidity on airborne pathogen viability and infectivity.

Pathogen type	Temperature	Relative humidity
Viruses	Decrease by higher temperature	Variable
Bacteria	Decrease by higher temperature	Variable
Fungi	Increase by higher temperature	Increase by higher relative humidity

**Table 6:** Summary of advantages and disadvantages of different viability models.

Viability model	Advantages	Disadvantages
Exponential	Simple, easy to fit, reasonable agreement with experiments	Underestimation of viability at long durations
Higher order kinetic	Physically plausible, good agreement with experiments	Difficult to fit
Catastrophe	Physically plausible, good agreement with experiments	Difficult to fit, too many model varieties

**Table 7:** Summary of advantages and disadvantages of different types of ventilation systems for hospitals [5].

Ventilation systems	Advantages	Disadvantages
Mechanical	Suitable for all climates and more controlled and comfortable environment	Expensive installation and maintenance, noisy, and not fail-safe
Natural	Suitable for warm climates, inexpensive, and capable of achieving high exchange rates	Difficult to predict actual performance, affected by outdoor conditions, reduced comfort level, and high-tech versions difficult to implement and control
Hybrid	Suitable for most climates, energy savings, and more flexible	May be expensive, and difficult to design and control

**Table 8:** Functional spaces in health care facilities [72].

Functional space category	Subspace functions (minimum required ACH±^1^)
Surgery and critical care	Class A (15+), B (20+), and C (20+) operations, newborn intensive care (6+), triage (12+)
Inpatient nursing	Patient recovery (6), protective environment (12+), airborne infection isolation (12−), corridor (2)
Skilled nursing facility	Resident (2), gathering/activity/dining (4)
Laboratories	Diagnostic radiology (6), surgical radiology (15+), bacteriology (6−), microbiology (6−), autopsy (12−), sterilizing (10−)
Diagnostic and treatment	Examination (6), medication (4+), treatment (6)
Sterilizing and supply	Sterilizing equipment (10−)
Central medical and surgical supply	Clean workroom (4+), sterile storage (4+)
Service	Food preparation (10), laundry (10−), bathrooms (10−)
Support	Hazardous material storage (10−)

^1^+: positive pressure required; −: negative pressure required.

**Table 9:** Supply diffuser types [72].

Diffuser type	Description
Group A	In or near ceiling, horizontal discharge
Group B	In or near floor, vertical nonspreading discharge
Group C	In or near floor, vertical spreading discharge
Group D	In or near floor, horizontal discharge
Group E	In or near ceiling, vertical discharge

**Table 10:** MERV Rating.

MERV	Typical controlled contaminant [*μ*m]	Dust spot efficiency [%]	Arrestance [%]
17–20	<0.3	NA^1^	NA
13–16	0.3–1.0	>90	>98
9–12	1.0–3.0	>40	>90
5–8	3.0–10	>20	>80
1–4	>10	>20	>65

^1^NA: not applicable.

**Table 11:** MERV Rating for health care functional spaces [72].

Space designation	Filter bank no. 1 (MERV)	Filter bank no. 2 (MERV)
Class B and C surgery rooms	7	14
Inpatient care, treatment, diagnosis, and airborne infection isolation rooms	7	14
Protective environment rooms	7	17 (HEPA)
Laboratories, class A surgery, and associated spaces	13	NR^1^
Administrative, food preparation spaces	7	NR
All other outpatient spaces	7	NR
Skilled nursing facilities	7	NR

^1^NR: not required.
